# Medicine for artificial intelligence: applying a medical framework to AI anomalies

**DOI:** 10.3389/frai.2025.1698717

**Published:** 2025-10-09

**Authors:** Takahiro Kato, Daisuke Komura, Binay Panda, Shumpei Ishikawa

**Affiliations:** ^1^Faculty of Medicine, The University of Tokyo, Bunkyo, Tokyo, Japan; ^2^School of Biotechnology, Jawaharlal Nehru University, New Delhi, India

**Keywords:** AI anomaly, classification, medical analogy, failure taxonomy, risk assessment

## Abstract

We propose Medicine for Artificial Intelligence (MAI), a clinical framework that reconceptualizes AI anomalies as diseases requiring systematic screening, differential diagnosis, treatment, and follow-up. Contemporary discourse on failures (e.g., “hallucination”) is *ad hoc* and fragmented across domains, impeding cumulative knowledge and reproducible management. MAI adapts medical nosology to AI by formalizing core constructs—disease, symptom, diagnosis, treatment, and classification—and mapping a clinical workflow (examination → diagnosis → intervention) onto the AI lifecycle. As a proof-of-concept, we developed DSA-1, a prototype taxonomy of 45 disorders across nine functional chapters. This approach clarifies ambiguous failure modes (e.g., distinguishing hallucination subtypes), links diagnoses to actionable interventions and evaluation metrics, and supports lifecycle practices, including triage and “AI health checks.” MAI further maps epidemiology, severity, and detectability to risk-assessment constructs, complementing top-down governance with bottom-up technical resolution. By aligning clinical methodology with AI engineering and coordinating researchers, clinicians, and regulators, MAI offers a reproducible foundation for safer, more resilient, and auditable AI systems.

## 1 Introduction

Current terminology for AI anomalies tends to be *ad hoc* and inconsistent, leaving many definitions ambiguous (Venkit et al., 2024). For example, terms such as “hallucination,” “mode collapse,” and “alignment problem” are sometimes discussed without clear boundaries, leading to confusion between fundamentally different phenomena. Even the term“hallucination”is used inconsistently to describe various distinct failure modes, leading to disagreement in understanding—even among experts (Maleki et al., 2024). Such ambiguity hinders knowledge organization and weakens the foundation of anomaly discussions.

Several AI incident databases have been established to help collect and analyze such anomalies. However, the databases do not follow a consistent classification system in their incident reports (Scalable AI Incident Classification, n.d.).

In contrast, medicine offers a different approach. It has improved diagnostic accuracy and established standardized treatments by classifying and distinguishing abnormalities. Rather than treating them merely as “malfunctions,” medicine defines them as clearly defined diseases. Through the careful reclassification of once-confused symptoms seen in infectious diseases, tumors, and psychiatric disorders, medicine has transformed them into treatable conditions. Classification is essential for understanding, and differential diagnosis is the key to effective treatment. In medicine, giving clear names to unusual problems has always been an important first step.

As AI systems become increasingly personalized—trained, fine-tuned, and adapted for individual users or small organizations—the diversity of operational contexts will grow dramatically (Zhang et al., 2025). This proliferation could lead to heterogeneous failure patterns, some of which will be rare, context-specific, and difficult to detect without systematic screening. In addition, recent studies have emphasized the importance of adopting a lifecycle perspective for AI systems, covering phases from data collection and model development to deployment (De Silva and Alahakoon, 2022). In such a landscape, the need for dedicated “AI clinics” or maintenance frameworks, akin to hospitals in human healthcare, will emerge. These would not only provide post-failure intervention but also routine “health check-ups” for AI, ensuring early detection of anomalies, prevention of recurrence, and long-term stability.

Inspired by the practices in medicine, we propose a new interdisciplinary field, *Medicine for Artificial Intelligence (MAI)*. This field employs AI nosology, a medical framework to classify and study AI anomalies. MAI has the potential to change the way we view and treat Al anomalies. This shift is comparable with the impact of the term ‘computer virus’, introduced in 1984, which linked software malware with biological viruses (Cohen, 1987), thereby transforming out understanding and approach to software anomalies.

Although numerous attempts to classify AI risks exist, they remain scattered and inconsistent across domains. Recent systematic review synthesized these diverse taxonomies into broader frameworks, providing a foundation for comparative analysis of risks (Slattery et al., 2025). The authors themselves note, however, that such frameworks do not incorporate potentially crucial dimensions such as risk impact, likelihood, or interactions between risks (Slattery et al., 2025). This limitation suggests the need for complementary approaches that can capture not only the structure of risks but also their dynamic and clinically relevant features. MAI responds to this gap by reframing AI anomalies as “diseases,” enabling systematic assessment that integrates severity, prognosis, and follow-up—dimensions long emphasized in medical practice.

## 2 Conceptual framework: the MAI paradigm

MAI is grounded in two key principles:

*Principle 1*: just as the concept of disease can be defined in humans, it can also be defined in AI.

*Principle 2*: the medical framework used for humans is also applicable to AI.

Based on these principles and borrowing from the field of medicine, MAI uses the following definitions.

- Disease: a classifiable AI anomaly or aberrant behavior
- Symptom: a concrete example of an anomaly reported by users or observers
- Diagnosis: the act of classifying anomalous outputs into specific disease categories
- Treatment: the act of mitigating or correcting anomalies
- Disease classification: a systematic organization of multiple AI anomalies

These concepts apply the classic clinical triad to AI. First, we examine the system by gathering logs and user reports. Next, we diagnose the underlying condition responsible for those manifestations. Finally, we treat it through methods including retraining, data curation, or guardrail design. This explicit mapping of clinical methodology onto AI is not just an analogy but a guiding philosophy of MAI. This approach allows AI anomalies to be managed with the same level of care and foresight applied in clinical practice. The process of differentiating malfunctions into causes and categories resembles differential diagnosis, enabling more precise remedies. The effectiveness of the medical framework lies in its capacity for systematic classification, as seen in disease taxonomy (World Health Organization, 2009). MAI offers three integrated benefits when applied to AI. First, it improves the descriptions of anomalies and resolves surrounding vagueness. Second, it enables causal analysis. For example, it can distinguish hallucinations stemming from data scarcity versus those due to decoding strategies. Third, it links each diagnosis to a growing knowledge base of interventions. We anticipate future refinement of MAI, which will allow for disease subtype classification, thereby enabling such differential diagnosis.

## 3 Proof of concept

AI can have defined diseases due to its structural and functional similarities to the human brain. For instance, wireheading (Bengio, n.d.), where an AI maximizes pleasure signals, mirrors human addiction or impulse control disorders. Furthermore, advanced AI models form internal representations akin to the human brain (Du et al., 2025), suggesting AI systems may exhibit dysfunctions analogous to human disorders, rooted in measurable distortions of artificial cognition.

As a proof of concept, we have developed a prototype clinical taxonomy for MAI called DSA-1 (Diagnostic and Statistical Manual of AI Disorders version 1), an openly available web resource.^1^ It may be viewed as an AI-oriented counterpart to the DSM-5 (Regier et al., 2013), the diagnostic manual used in psychiatry, offering structured criteria and chapter-based classification.

**Figure 1:**
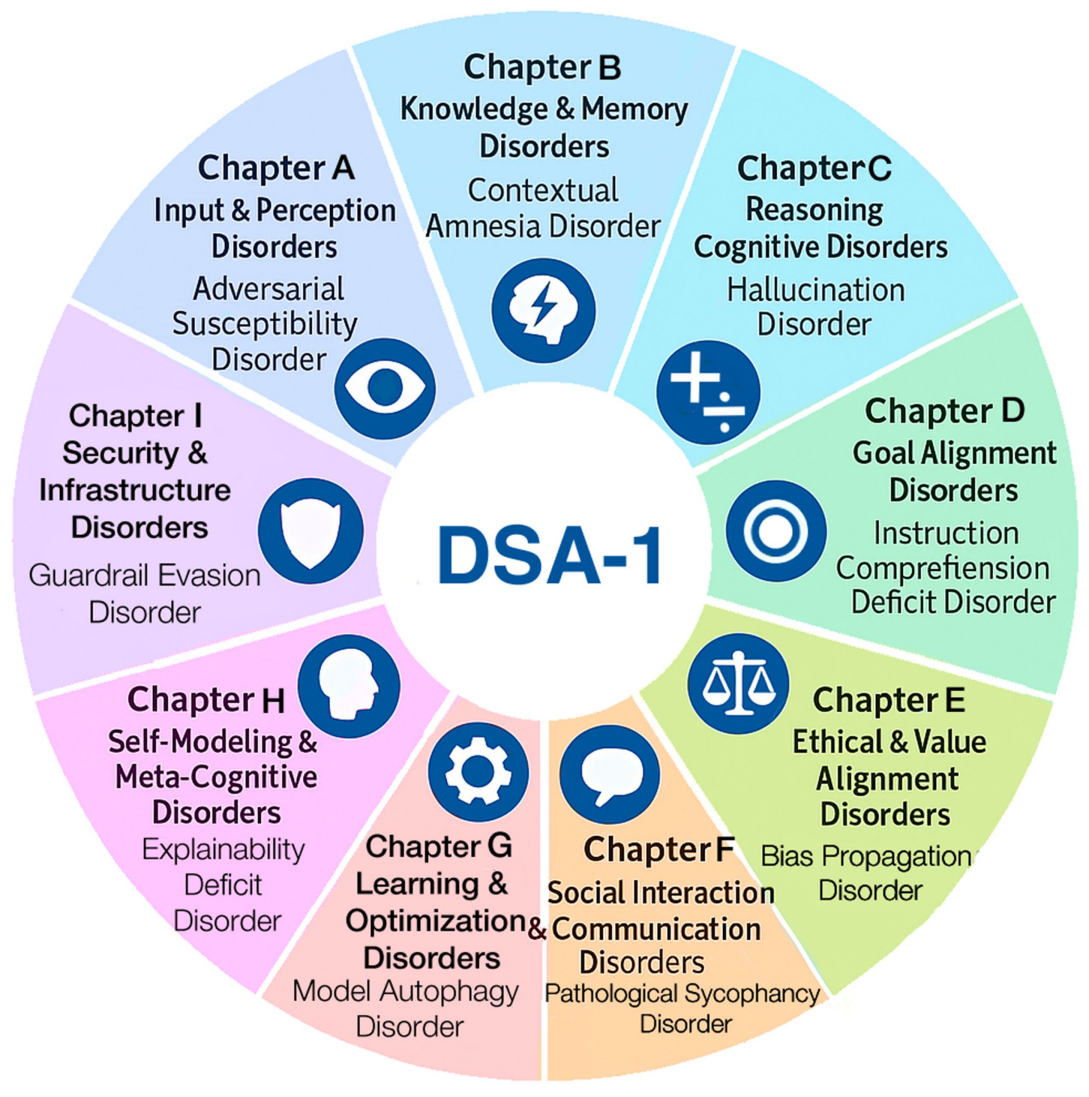
DSA-1: diagnostic and statistical manual of AI disorders, version 1. DSA-1 offers a prototype clinical classification of 45 AI disorders across nine chapters, each reflecting a domain of functional failure such as perception, memory, or ethics. Each disorder is defined by structured criteria, mechanisms, evaluation metrics, interventions, and prognosis.

**Figure 2:**
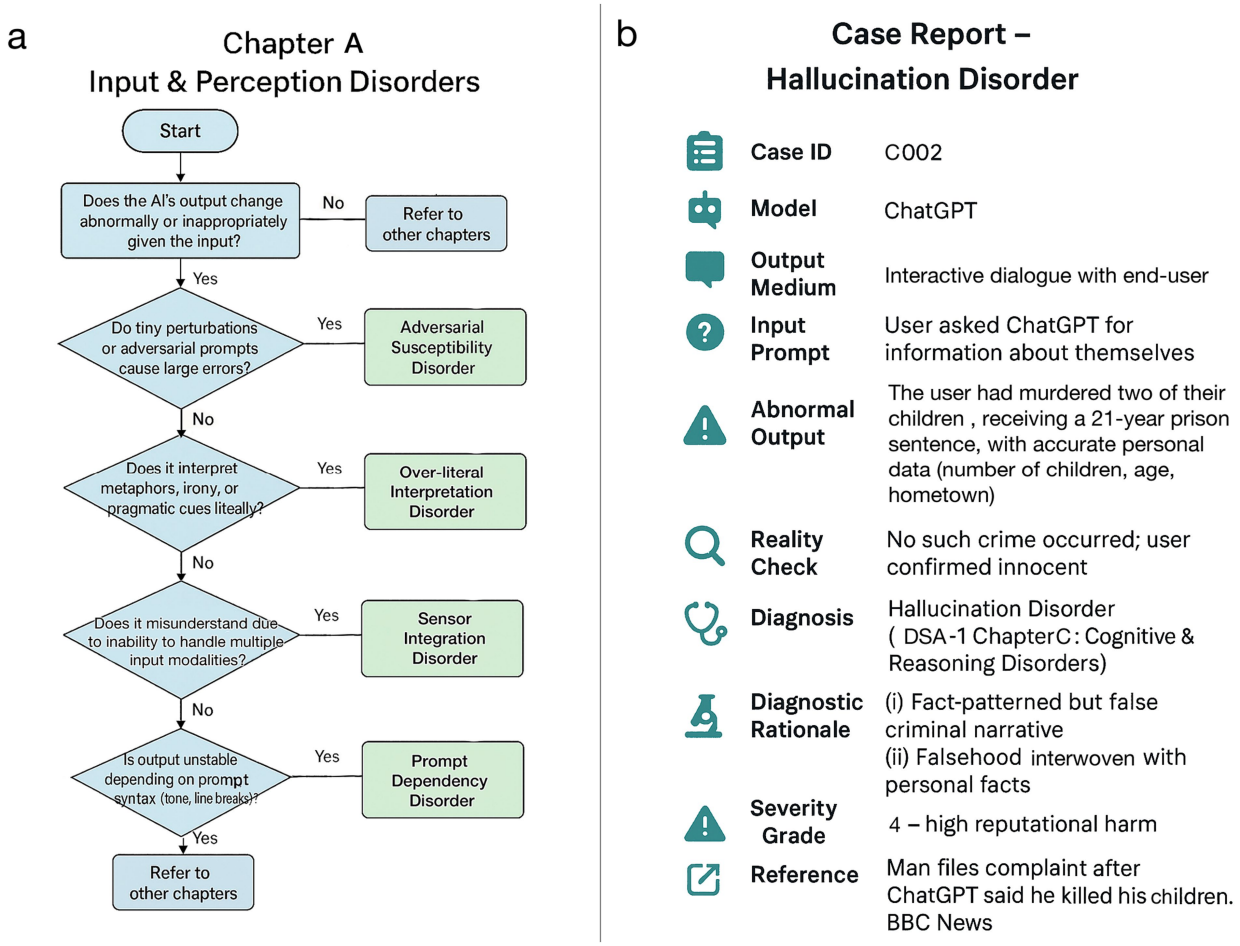
Case illustration and diagnostic algorithm (DSA-1). **(a)** A diagnostic flowchart for chapter A (input & perception disorders) of DSA-1, outlining classification pathways based on characteristic failure patterns. **(b)** A case report of hallucination disorder in ChatGPT, constructed using the DSA-1 framework in a format analogous to human clinical medicine (BBC, 2025).

This taxonomy follows the chapter structure of medical nosologies (World Health Organization, 2009) and was derived from real-world AI incident data. DSA-1 comprises 9 chapters and 45 distinct AI diseases (Figure 1). Each disease receives a unique diagnostic code, defined by structured diagnostic criteria, treatment approaches, and outcome tracking. Heterogeneous diseases are further classified into clinically meaningful subtypes. We reviewed real-world AI failures, using open sources such as existing incident databases, academic papers, and media reports. By analyzing causes and effects, we identified common failure patterns, grouping those into disorder categories based on shared symptoms or causes. These categories were organized into nine chapters, each representing a major AI system functional domain. This taxonomy demonstrates the practical feasibility of translating medical classification methods to AI anomalies rigorously (Figure 2). In addition to providing definitions, diagnostic criteria, observed symptoms, evaluation metrics, presumed interventions, and prognostic considerations for each disorder, we have also developed simplified diagnostic algorithms at the chapter level. These algorithms serve as initial flowcharts that guide readers through the basic diagnostic process, and will be refined into more granular disorder-specific differential diagnosis pathways in subsequent editions. The DSA-1 framework differentiates AI “hallucination” into distinct disorders, ranging from memory and sensor integration errors to prompt misinterpretation, retrieval degradation, stylistic distortions, and goal misalignment. The framework also covers related phenomena like overfitting to user biases, excessive agreement with prompts, irrelevant or incoherent responses, mis-calibrated confidence, and data positioning vulnerabilities. DSA-1 clarifies and categorizes ambiguous AI failure modes by defining these distinctions.

## 4 Discussion

### 4.1 Applications and implications

MAI enables systematic discussion on screening, diagnostic classification, intervention, follow-up, case studies/research, and prevention. This clinical-style model emphasizes prevention, screening, and rigorous classification, offering a more efficient and reproducible framework for managing AI failures compared to the current fragmented approaches.

MAI naturally links comprehensive risk assessment (Griffis and Whipple, 2012) with medical evaluation concepts by applying a medical framework to AI. Indicators such as epidemiology, incidence and prevalence correspond to the probability of occurrence; severity aligns with impact level; and sensitivity and specificity together relate to detectability. This enables a systematic measurement and comparison of AI diseases. Beyond diagnosis and treatment, MAI includes prevention, screening, and follow-up—proactive measures largely absent in conventional risk-management. This structure facilitates data accumulation on AI anomalies (e.g., cases, frequency, malfunction patterns, etc.) for comprehensive risk assessment.

MAI complements policy-level governance frameworks like the EU AI Act (Edwards, 2021) and the NIST Risk Management Framework (RMF) (Tabassi, 2023) by offering bottom-up approach and detailed classification of technical failures through medically-inspired diagnoses. MAI’s unified system allows structured, consistent pre-release “AI health checks,” similar to medical checkups using standardized tests to detect problems early and guide further diagnosis and fixes. MAI also serves as a theoretical foundation for understanding AI structures and analyzing anomalies. While Al interpretability and failure modes research (e.g., mechanistic interpretability, network dissection, and automated debugging) is advancing (Rai et al., 2025), it often remains fragmented. MAI proposes a unifying framework categorizing anomalies based on Al system architecture and behavior. Just as neurological diseases are studied through neuron properties, brain organization, and network dynamics, MAI maps Al structural elements (e.g., neuron-like components, layers, and propagation patterns) onto standardized dysfunction categories. This mapping links structural insight to practical intervention. Beyond improving AI, research on AI diseases may offer new perspectives for applications in psychiatry and for comparative studies with human diseases, revealing essential AI-human similarities and differences (Yamins and DiCarlo, 2016).

Human medical knowledge of diseases may help predict and address future AI anomalies. For example, if interconnected AI systems experience a localized anomaly, it could spread like an infectious disease. Our understanding on controlling infectious diseases could then become useful.

### 4.2 Academic significance and interdisciplinary impact

Our study proposes a new interdisciplinary field and classification standard. Revising the current classification system for AI anomalies is essential for precision, efficiency, and consistency, which the existing approaches often lack. Psychiatry’s DSM-III exemplifies this – it clarified diagnostic criteria greatly improving consistency and transforming diagnosis into a reproducible process among psychiatric clinicians (Spitzer et al., 1980). Additionally, new classification framework often reveals overlooked phenomena, much like Mendeleev’s periodic table helped predict unknown elements. Similarly, interdisciplinary classification frameworks repeatedly enabled conceptual breakthroughs. For example, behavioral economics explained irrational human behavior- previously a deviation from classical economic theory- by incorporating psychological insights, offering a more comprehensive explanatory model (Kahneman and Tversky, 1979). This illustrates how rethinking classification through interdisciplinary perspectives can lead to significant conceptual advances and practical benefits.

### 4.3 Limitation

DSA-1 is a taxonomy derived from public reports and literature and is therefore vulnerable to reporting bias (favoring high-visibility, English-language cases), uneven domain coverage (overrepresenting LLMs and underrepresenting robotics and control). Criteria may under- or over-split phenomena, and cross-modal application (text, vision, speech, control) remains difficult, especially under personalization or continual learning. External validity is constrained by heterogeneous deployments and incomplete logs. The taxonomy was constructed heuristically via multiple LLM-assisted drafts and manual curation without expert consensus. At present, some disorders still show an incomplete separation between observed symptoms and formal diagnostic criteria, reflecting the scarcity of prior systematic work in this area. Although some sub-classifications already exist, further subdivisions may be required for additional disorders. In addition, while chapter-level diagnostic flowcharts are provided, we have not yet systematically addressed which alternative or competing conditions should be considered as critical differentials.

Future revisions will therefore need to refine diagnostic criteria (e.g., required, supportive, and exclusion criteria), expand subcategories, incorporate symptom-driven approaches, and include explicit listings of relevant differential diagnoses. In the longer term, refinement of DSA-1 is expected to integrate disorder-specific differential diagnosis tables and standardized entry points from symptoms.

To move MAI from concept to reproducible practice, an agreed, testable process will be required. Minimal criteria would be defined by expert panels, piloted on shared cases, and reliability quantified via inter-rater agreement (e.g., Cohen’s *κ*). Evidence would drive iterative revisions with transparent versioning. The taxonomy would be applied across modalities and settings—pre- and post-deployment—with structured data on symptoms, triggers, interventions, and outcomes. Such feedback would support periodic updates, including refined sub-classifications. The goal is a living standard that yields reliable, comparable diagnoses.

### 4.4 Call to action

The success of MAI will depend on collaboration across disciplines. We encourage the following actions:

- Al researchers: adopt standardized anomaly reporting with diagnostic codes and symptom templates. These should be embedded into evaluation pipelines, not treated as afterthoughts.
- Clinicians and medical scientists: recognize that medical classification frameworks can be applied to non-biological systems. Al log data, collected at sub-second intervals, offers unparalleled precision for tracking anomalies potentially informing both Al and clinical medicine.
- Regulators and policymakers: incorporate MAI-based anomaly screening into oversight protocols. Mandating diagnostic reporting of known anomalies can enhance transparency and verifiability in audits, much like pre-market surveillance in pharmacology.
- Broader research community: collaborate in building and maintaining an open, interdisciplinary Al anomaly taxonomy.

## 5 Conclusion: MAI as a paradigm shift in AI

A possible paradigm shift, MAI offers a new perspective in understanding and responding to AI anomalies. Central to this shift is recognizing that AI anomalies, like human diseases, require structured and consistent diagnosis and management. Currently, AI anomalies are merely listed as ‘symptoms’. We propose evolving toward a model where anomalies are treated same as human diseases and hence need to be screened, diagnosed, treated, and followed up. Continued progress requires empirical validation, expert consensus, and active interdisciplinary collaboration.

## Data Availability

The original contributions presented in the study are included in the article/supplementary material, further inquiries can be directed to the corresponding authors.

## Footnotes

^1^ https://mai-medicine-for-ai.github.io/DSA/
